# Breast cancer histopathology image classification through assembling multiple compact CNNs

**DOI:** 10.1186/s12911-019-0913-x

**Published:** 2019-10-22

**Authors:** Chuang Zhu, Fangzhou Song, Ying Wang, Huihui Dong, Yao Guo, Jun Liu

**Affiliations:** 1 The Center for Data Science, the Beijing Key Laboratory of Network System Architecture and Convergence, the School of Information and Communication Engineering, Beijing University of Posts and Telecommunications, Xitucheng Road, Beijing, China; 2 The Department of Pathology, Beijing Chaoyang Hospital, the Third Clinical Medical College of Capital Medical University, Gongren Tiyuchang Nanlu, Beijing, China

**Keywords:** Breast cancer, Channel pruning, Histopathology, Hybrid CNN

## Abstract

**Background:**

Breast cancer causes hundreds of thousands of deaths each year worldwide. The early stage diagnosis and treatment can significantly reduce the mortality rate. However, the traditional manual diagnosis needs intense workload, and diagnostic errors are prone to happen with the prolonged work of pathologists. Automatic histopathology image recognition plays a key role in speeding up diagnosis and improving the quality of diagnosis.

**Methods:**

In this work, we propose a breast cancer histopathology image classification by assembling multiple compact Convolutional Neural Networks (CNNs). First, a hybrid CNN architecture is designed, which contains a global model branch and a local model branch. By local voting and two-branch information merging, our hybrid model obtains stronger representation ability. Second, by embedding the proposed Squeeze-Excitation-Pruning (SEP) block into our hybrid model, the channel importance can be learned and the redundant channels are thus removed. The proposed channel pruning scheme can decrease the risk of overfitting and produce higher accuracy with the same model size. At last, with different data partition and composition, we build multiple models and assemble them together to further enhance the model generalization ability.

**Results:**

Experimental results show that in public BreaKHis dataset, our proposed hybrid model achieves comparable performance with the state-of-the-art. By adopting the multi-model assembling scheme, our method outperforms the state-of-the-art in both patient level and image level accuracy for BACH dataset.

**Conclusions:**

We propose a novel compact breast cancer histopathology image classification scheme by assembling multiple compact hybrid CNNs. The proposed scheme achieves promising results for the breast cancer image classification task. Our method can be used in breast cancer auxiliary diagnostic scenario, and it can reduce the workload of pathologists as well as improve the quality of diagnosis.

## Background

Breast cancer has high morbidity and mortality among women according to the World Cancer Report [1], and this type of cancer causes hundreds of thousands of deaths each year worldwide [2]. The early stage diagnosis and treatment can significantly reduce the mortality rate [3]. The histopathological diagnosis based on light microscopy is a gold standard for identifying breast cancer [4]. To conduct breast cancer diagnosis, the materials obtained in the operating room are first processed by formalin and then embedded in paraffin [5]. After that, the tissue is cut by a high precision instrument and mounted on glass slides. To make the nuclei and cytoplasm visible, the slides are dyed with hematoxylin and eosin (HE). Finally, the pathologists finish diagnosis through visual inspection of histological slides under the microscope. However, the histopathological examination requires the pathologists having a strong professional background and rich experience, and the primary-level hospitals and clinics suffer from the absence of skilled pathologists [6]. Besides, the traditional manual diagnosis needs intense workload, and diagnostic errors are prone to happen with the prolonged work of pathologists.

One possible solution to address the above problems is designing intelligent diagnostic algorithm. It can learn from the senior pathologists and then inherit the experience, which can be used to train the young pathologists. Besides, with the help of powerful computing ability of hardware, such as GPU, the automatic algorithm can speed the manual diagnosing process and reducing the error rate.

Extensive pieces of literature [7–12] design automatic breast cancer histopathology image recognition schemes. Typically, the algorithms of the literature can be classified into two categories. In the first category, nuclei segmentation is performed and then hand-crafted features, such as morphological and texture features, are extracted from the segmented nuclei. Finally, the generated features are put into classifiers for automatic image type decision [7–9]. In work [9], the authors introduce a large, publicly available and annotated dataset, which is composed of 7909 clinically representative, microscopic images of breast tumor tissue images collected from 82 patients. Six hand-crafted features, such as LBP [13] and LPQ [14], and 4 traditional classifiers, such as 1-Nearest Neighbor (1-NN) and Support Vector Machines (SVM), have been comprehensively evaluated. Generally, great efforts and effective expert domain knowledge are required to design appropriate features for this type of method.

In the second category, different Convolutional Neural Networks (CNNs) are adopted to recognize histopathology image [10–12]. The recent research shows that CNN-based algorithms achieve promising results, which outperform the best traditional machine learning method. The authors in [15] introduce deep learning to improve the analysis of histopathologic slide and conclude that it holds great promise in increasing diagnosis efficacy. In work [16], the authors use deep max-pooling CNN to detect mitosis, which is an important indicator of breast cancer. The proposed method won the ICPR 2012 mitosis detection competition. In order to save the training time, the DeCAF features are extracted by using a pre-trained CNN and then a classifier is learned for the new classification task [10]. Both single task CNN and multi-task CNN architectures are proposed to classify breast cancer histopathology images [17]. Most of the CNN-based schemes in the second category just adopt one single model to recognize cancer, the generalization ability is insufficient. The authors of work [11] train different patch-level CNNs and merge these models to predict the final image label based an improved existing CNN, and achieves state-of-the-art results on the large public breast cancer dataset [9].

Although the above CNN-based methods achieve better results than the first category, the used networks generally have more model parameters and higher computing burden in inference stage, and thus they are more complex than the traditional scheme. Especially, the recently designed networks tend to have more layers and parameters, such as the ILSVRC 2015 winner ResNet [18] has more than 100 layers and 60 million parameters. This will cause several problems: big store space requirement, large run-time memory consumption during inference, higher classification latency due to the millions of computing operations.

To address these problems, many works have been proposed to compress large CNNs for fast inference [19–26]. The authors in [23] propose a HashedNets architecture, which can exploit inherent redundancy in neural networks to achieve reductions in model size. HashedNets uses a low-cost hash function to randomly group connection weights into hash buckets, and all connections within the same hash bucket share the same parameter value. Although the storage space can be reduced by this kind of architecture, neither the run-time memory nor the inference time can be decreased. In [24], a three-stage compression pipeline is proposed: prune the important connections of the network, then achieve weight sharing by quantizing the weights, and finally apply Huffman coding to further remove the redundancy. This method achieves remarkable results on model size compression and time saving, but many different techniques need to be applied together. A dynamic and more efficient method is proposed to prune neural network weights in [25]. However, it needs specially designed software or hardware accelerators to reduce run-time memory and inference time. Recently, the authors in [26] propose a network slimming scheme to achieve channel-level sparsity in deep CNNs. They directly use the specific parameter of BN layers as the channel scaling factor to identify and remove the unimportant channels during training. However, the adopted parameter does not explicitly model interdependencies between channels and thus the channel importance is not decently extracted.

Most of the above model compression methods can only address one or two challenges mentioned above and some of the techniques require specially designed software/hardware accelerators [25]. Besides, few deep model compression studies pay attention to the breast cancer histopathology dataset.

Two important challenges are left open in the existing breast cancer histopathology image classification:

- The adopted deep learning methods usually design a patch-level CNN, and put the downsampled whole cancer image into the model directly. However, due to the information loss introduced by the downsampling, the models are not sufficient to capture the local detail information. The model with stronger representation which can extract both global structural information and local detail information simultaneously is worth studying.
- The larger CNNs produce stronger representation power, but consume larger on-chip/off-chip memory and utilize more computing resource, which leads to higher diagnosing latency in many real-world clinical applications. How to design a compact yet accurate CNN to alleviate the problems is still challenging.

In this work, we propose a breast cancer histopathology image classification through assembling multiple compact CNNs to address the above two challenges.

The contributions of this paper are summarized in the following:

- A hybrid CNN architecture is designed, which contains a global model branch and a local model branch. By local voting and two-branch information merging, our hybrid model obtains stronger representation ability.
- To alleviate the effect of large model size and generate compact CNN, we first propose the Squeeze-Excitation-Pruning (SEP) block based on the original Squeeze-Excitation (SE) module in [27], and then embed it into the hybrid model. Thus the channel importance can be learned and the redundant channels are removed.
- To further improve the generalization ability of classification, we further propose a special model bagging scheme. Multiple models are built with different data partition and composition, and then they are assembled together to vote for the final result.

## Methods

In this section, we propose our breast cancer histopathology image classification scheme. Firstly, we introduce the proposed hybrid CNN architecture and local/global branches. Then, we present the preprocessing, dataset augmentation and the compact CNN model design flow, and finally, model assembling will be described.

### Hybrid cNN architecture

**Fig. 1:**
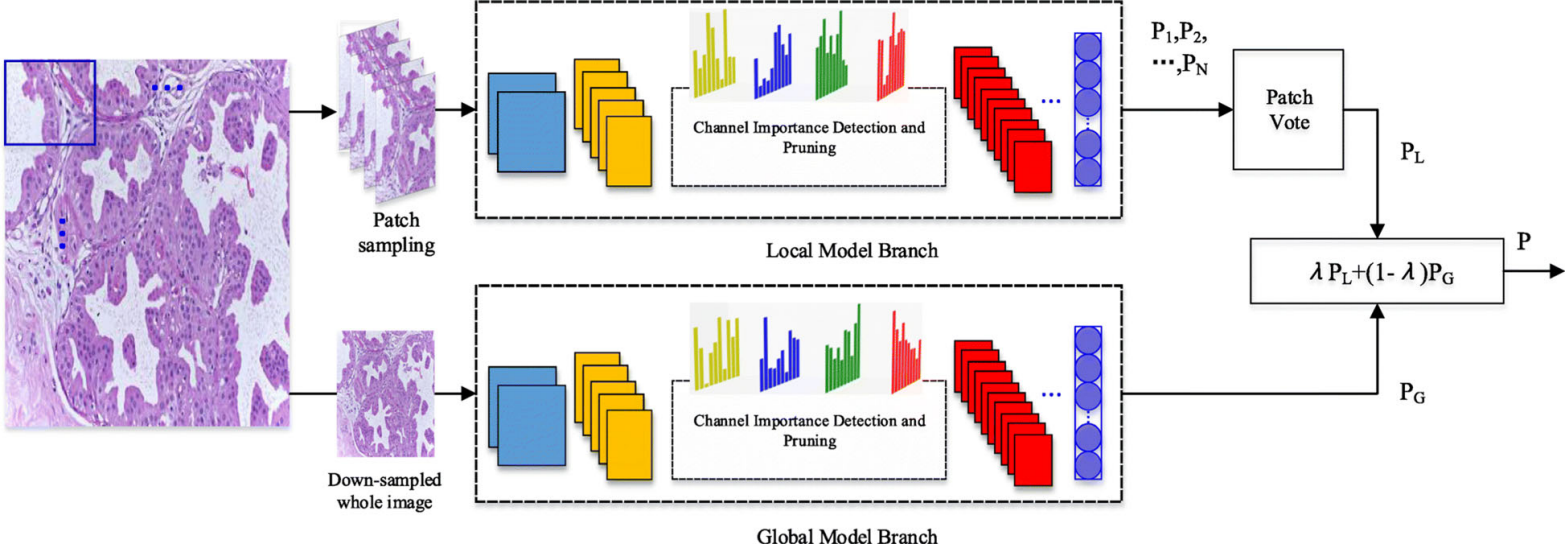
Proposed hybrid CNN architecture. Two model branches are integrated together to extract more key information, and the channel pruning module is embedded to compact the network

To merge more key information when in classification, a hybrid CNN unit is proposed. The proposed framework of our hybrid CNN architecture is shown in Fig. 1. It mainly includes a local model branch and a global model branch. For a histopathology image, on the one hand, a patch sampling strategy is performed first and a series of image patches are generated. Then the produced patches are passed to the local model branch, and *N* predictions (*P*_1_,*P*_2_,...,*P*_*N*_) are yielded for the *N* image patches. Patching voting is further performed for the *N* predictions and thus the final output *P*_*L*_ for the local model branch is generated. On the other hand, the downsampled input image as a whole is put into the global model branch and the prediction *P*_*G*_ is obtained. Finally, the local prediction *P*_*L*_ and the global prediction *P*_*G*_ are weighted together by *λ*, as shown in (1).

1 $$\begin{array}{@{}rcl@{}} {P}= {\lambda} P_{L} + {(1-\lambda)} P_{G}  \end{array} $$

### Global/Local model branch

**Fig. 2:**
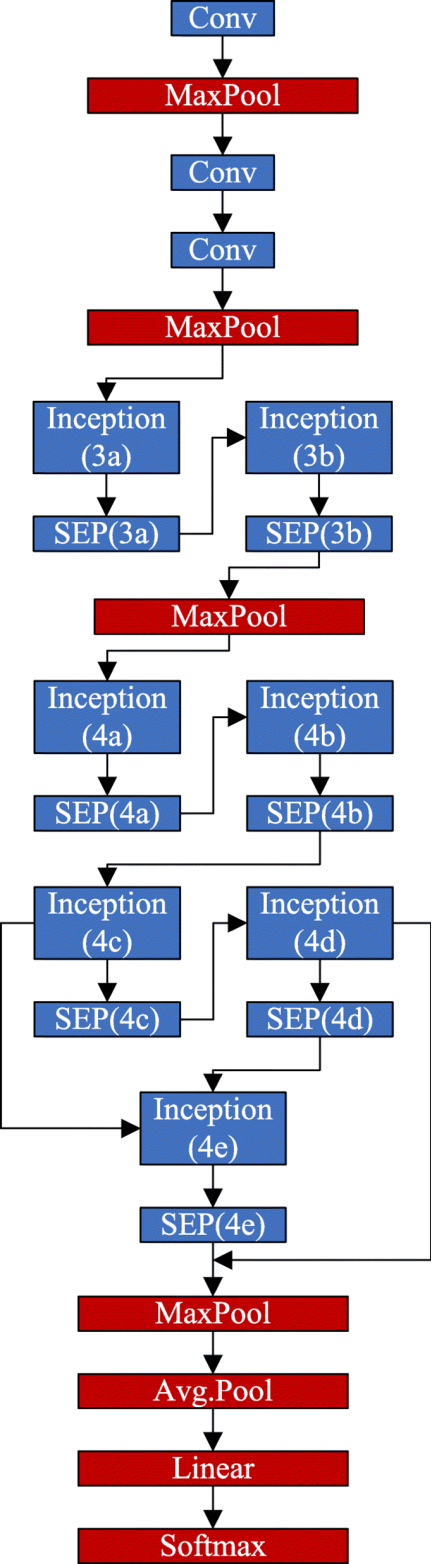
The designed CNN architecture. Both the global branch and the local branch adopt the same structure

The global and local model branch adopt the same CNN structure, as shown in Fig. 2. Table 1 illustrates the details of our proposed CNN.

**Table 1:** The details of the proposed initial CNN model

Type	**Patch size/Stride**	**Output**	**Depth**	**Params**
Convolution	7 ×7/2	112 ×112×64	1	2.7K
Max pool	3 ×3/2	56 ×56×64	0	
Convolution	1 ×1/1	56 ×56×64	1	0.8K
Convolution	3 ×3/1	56 ×56×192	1	112K
Max pool	3 ×3/2	28 ×28×192	0	
Inception(3a)		28 ×28×256	2	159K
SEP block	1 ×1	28 ×28×256	2	32K
Inception(3b)		28 ×28×480	2	380K
SEP block	1 ×1	28 ×28×480	2	32K
Max pool	3 ×3/2	14 ×14×480	0	
Inception(4a)		14 ×14×512	2	364K
SEP block	1 ×1	14 ×14×512	2	32K
Inception(4b)		14 ×14×512	2	437K
SEP block	1 ×1	14 ×14×512	2	32K
Inception(4c)		14 ×14×512	2	840K
SEP block	1 ×1	14 ×14×512	2	32K
Inception(4d)		14 ×14×528	2	580K
SEP block	1 ×1	14 ×14×528	2	32K
Inception(4e)		14 ×14×1856	2	840K
SEP block	1 ×1	14 ×14×1856	2	32K
Max pool	3 ×3/2	7 ×7×1856	0	
Ave pool	7 ×7/1	1 ×1×1856	0	
Linear		1 ×1×2	1	2K
Softmax		1 ×1×2	0	

The output of the convolution layer and SEP block may change after the channel pruning stage in every model compression loop

**Fig. 3:**
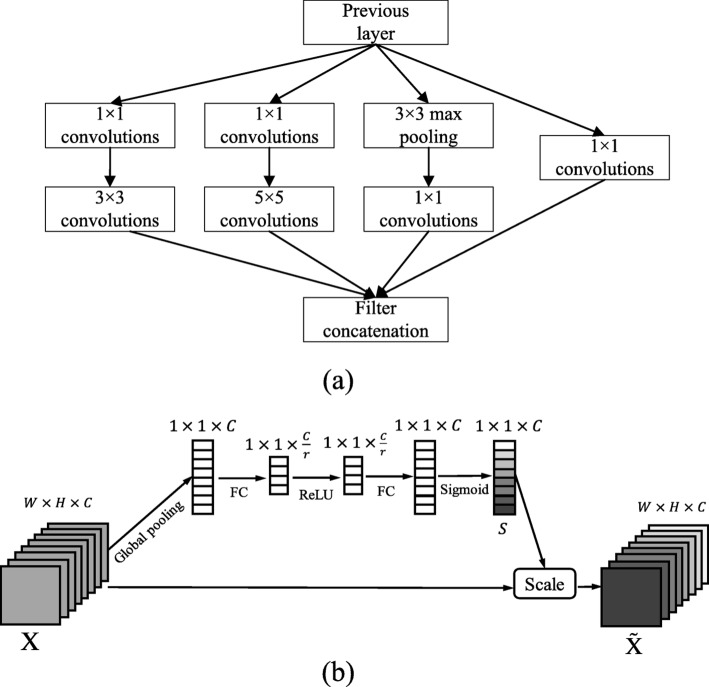
(**a**) Adopted inception architecture. (**b**) The basic structure of the SE block

In our work, the Inception module [28], residual network [18], and Batch Normalization (BN) techniques [29] are combined together to ensure recognition performance. The adopted Inception architecture is composed of a shortcut branch and a few deeper branches, as shown in Fig. 3(a). The Inception network consists of 1 ×1, 3 ×3, 5 ×5 filters, and 3 ×3 max pooling. In the structure, 1 ×1 convolutions are used to compute reductions before the expensive higher dimensional filters: 3 × 3 and 5 ×5 convolutions. In our model, totally seven Inception layers are integrated to address the problem of gradients vanishing/exploding, which guarantees the performance of deeper models. To further gain accuracy from considerably increased depth and to make our model easier to optimize, we adopt residual networks (Inception-4c to Inception-4e, Inception-4d to SEP-4e) in the model. Besides, the BN technique is adopted to allow the utilization of much higher learning rates and be less careful about initialization by normalizing layer inputs, which ensures a high robustness of our model.

As shown in Fig. 2, we connect each Inception module to a SEP block, which is used to compress our model. The proposed SEP block is constructed based on the original SE block in work [27] by adding the channel pruning power. The SE block can adaptively recalibrate channel-wise feature responses by explicitly modeling interdependencies between channels. The basic structure of the SE block is illustrated in Fig. 3(b). For feature maps **X**∈*R*^*W*×*H*×*C*^ of the CNN layer (e.g. the Inception module), they are first passed through a squeezing operation, which aggregates the feature maps across spatial dimensions *W*×*H* to produce a 1×1×*C* channel descriptor. The squeezing operation is implemented by a global pooling, and the channel descriptor embeds the distribution of channel-level feature responses. After global pooling, a statistic vector **z**∈*R*^*C*^ is generated [27]. **z**=[*z*_1_,...,*z*_*i*_,...,*z*_*C*_], and the *i*-th element of **z** is calculated by:

2 $$\begin{array}{@{}rcl@{}} {z_{i}}= \frac{1}{H \times W}\sum_{m=1}^{H} {\sum_{n=1}^{W}{x_{i}(m,n)}}  \end{array} $$

Then an excitation operation is performed on the generated channel descriptor to learn the sample-specific activation factor **s**=[*s*_1_,*s*_2_,...,*s*_*C*_] for *C* channels by using two fully-connected (FC) layers and two corresponding activation layers (ReLu and Sigmoid). The excitation operation can explicitly model interdependencies between channels. According to [27], **s** can be denoted as:

3 $$\begin{array}{@{}rcl@{}} \textbf{s} = \sigma(\textbf{W}_{2})\delta(\textbf{W}_{1}\textbf{z}))  \end{array} $$

where *δ* and *σ* are activation functions ReLu and Sigmoid for the two FC layers, respectively; $\textbf {W}_{1}\in R^{\frac {C}{r} \times C}$ and $\textbf {W}_{2}\in R^{C \times \frac {C}{r}}$ (in this work *r*=16) are weights of the two FC layers. Then the feature maps **X** are reweighted to $\tilde {\textbf {X}}$ :

4 $$\begin{array}{@{}rcl@{}} \tilde{\textbf{X}}= \textbf{s} \cdot \textbf{X} = \left[{s_{1}}\cdot \textbf{x}_{1},{s_{2}}\cdot \textbf{x}_{2},...,{s_{C}}\cdot \textbf{x}_{C}\right]  \end{array} $$

where $\tilde {\textbf {X}} = \left [\tilde {\textbf {x}}_{1},\tilde {\textbf {x}}_{2},...,\tilde {\textbf {x}}_{C}\right ]$, and **X**=[**x**_1_,**x**_2_,...,**x**_*C*_].

**Fig. 4:**
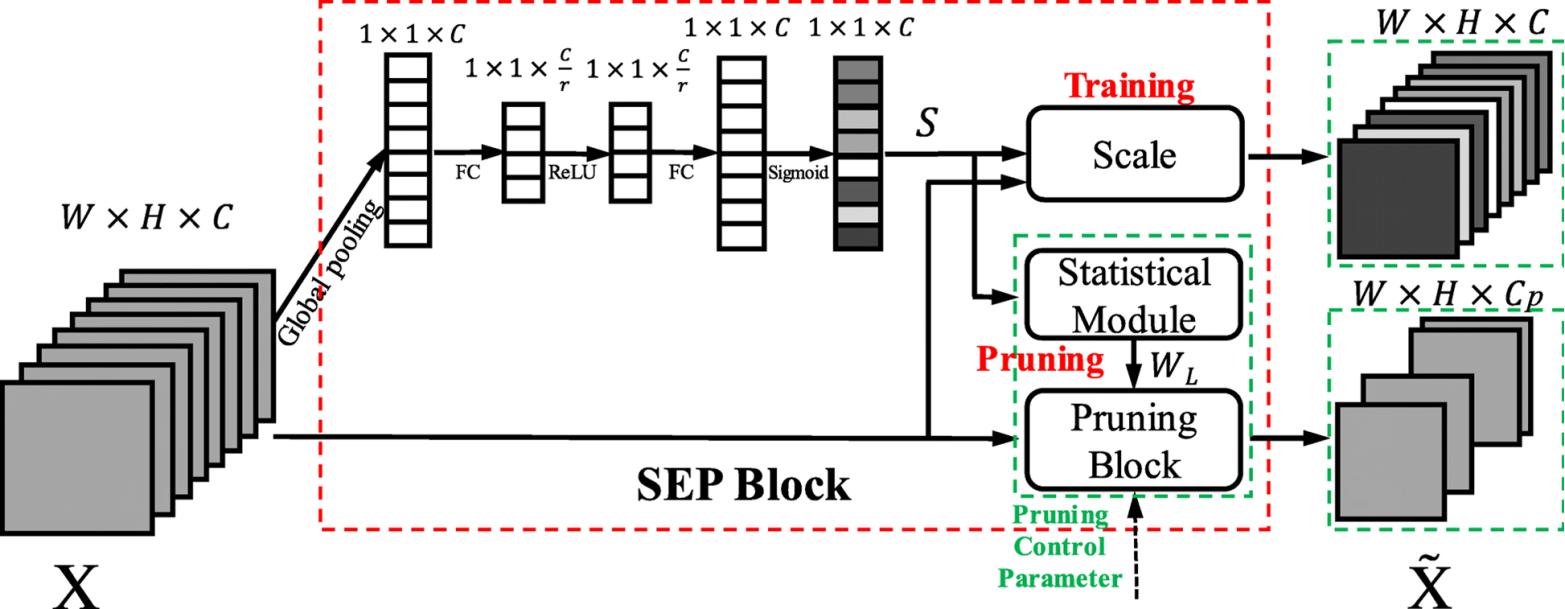
The proposed SEP block. The SEP block contains the original Scale, the added Statistical Module and Pruning Block. In the training stage, the original SE network is learned with Scale operation; in the pruning stage, the channel importance is obtained in Statistical Module and Pruned by using Pruning Block

In our work, we use the activation factors *s*_*i*_ (*i*=1,2,...,*C*) obtained by SE block as channel weights in assisting the model compression. Through embedding the statistical module and pruning block, our proposed SEP block can realize channel pruning function, as shown in Fig. 4. Specifically, the SEP block works differently in the training stage and pruning stage. In the training stage, the SEP performs like the original SE block: the *C* channels are connected to the scale module and then reweighted. The original SE part is trained within the entire network. In the pruning stage, the SEP block first makes statistics on the activation factors for all the training samples. Then it derives the channel weights *W*_*L*_ (taking Layer *L* for example) for the entire training dataset. Finally, the channel-level pruning will be performed according to the pruning control parameter, and the original *C* channels will be compressed to *C*_*p*_ channels. The detailed channel pruning process will be discussed in compact model design part.

Besides the Inception layers and SEP blocks, the convolution layers with size 1 ×1, 3 ×3 and 7 ×7 are used in our model.

### Preprocessing and dataset augmentation

#### Preprocessing

The breast histology microscopy we used in our work is stained by HE, and this staining method can help medical workers better observe the internal morphology of the tissue cells. However, color variation happens due to differences in staining procedures, and these color differences of the histology images may adversely affect the training and inference process in CNNs. We adopt the image processing methods in [30], which presents an approach for a more general form of color correction. This method uses a simple statistical analysis to impose the color characteristics of one image on another, and thus can achieve color correction by choosing an appropriate source image.

#### Dataset Augmentation

To avoid the risk of overfitting, data augmentation is often performed for the training process after dataset splitting. The strategies we used include random rotation, flipping transformation and shearing transformation. Unlike the augmentation methods (rotation with fixed angles) in [12], we rotate the images randomly. Besides, the shearing transformation method is also used, which zooms in or zooms out images in different directions. For each training sample, eight images are generated by using our adopted data augmentation method.

### Compact model design

**Fig. 5:**
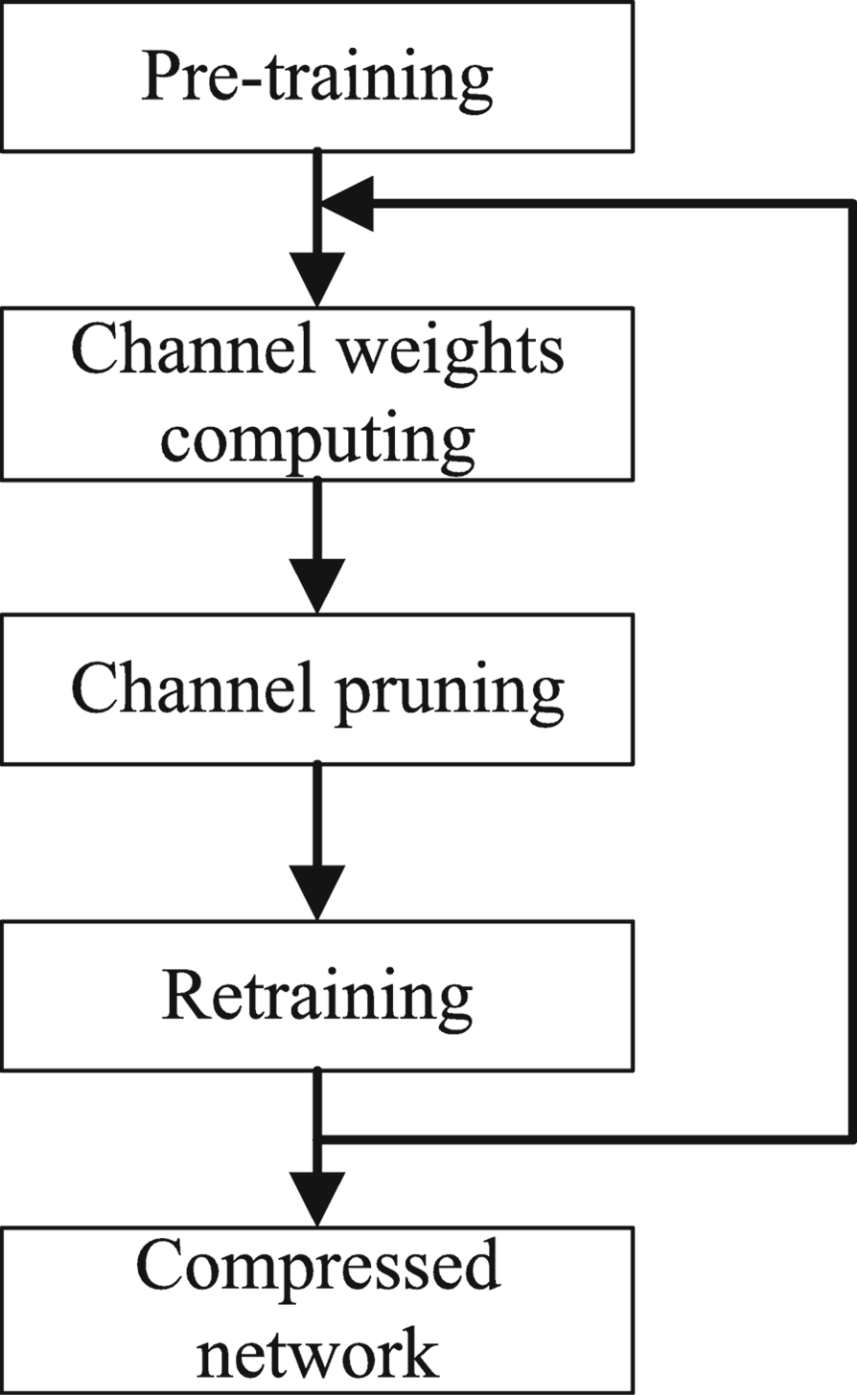
Channel pruning flow

The hybrid CNN architecture proposed above is pre-trained first. In this section, we will conduct model compression based on the pre-trained model and thus remove the model redundancies by channel pruning. The pruning flow is shown in Fig. 5. First, based on the pre-trained initial network, the channel weights are calculated by using the embedded SEP block. Then the unimportant channels with lower weights are discarded to make the network compact. After that, the newly compressed network is retrained to guarantee the high accuracy on the dataset. The three steps are repeated for several loops before finishing the model compression process. The channel weights computing and channel pruning will be detailed in the following.

#### Channel weights computing

After the retraining process in the previous loop, the model weights of FC layers in the SEP subnetwork are re-generated. We should notice that for the first pruning loop, the related weights are produced by the initially pre-trained network. By using these model weights and the corresponding activation layers, the *C* activation factors *s*_1_, *s*_2_,..., *s*_*C*_ corresponding to *C* channels of one layer can be calculated. Generally, the key channels to the final classification results are prone to have higher activation factors and vice verse. Thus the activation factors are chosen as channel weights for model compression. For each training sample, the corresponding sample-specific channel weights can be produced. Then the question is how to evaluate the entire channel importance for our model based on thousands of training samples. For each channel of the model, the channel-weight average on the training set is directly selected as its importance measure.

Suppose that the size of the training set is *N*. For a CNN with *M* convolutional layers, a specific convolution layer *L*_*D*_ (*D* from 1 to *M*) has *C* channels. Corresponding to the *C* channels, the channel importance is denoted as $W_{L_{D}} = \left [w_{D1}, w_{D2},..., w_{DC}\right ]$. For training sample *T*_*j*_ (*j* from 1 to *N*), the channel activation factors are [*s*_*D*1*j*_,*s*_*D*2*j*_,...,*s*_*DCj*_], thus the channel importance for layer *L*_*D*_ can be described as

5 $$ \begin{aligned} W_{L_{D}} &= \left[w_{D1}, w_{D2},..., w_{DC}\right] \\ &= \left[\frac{\sum_{j=1}^{N} s_{D1j}}{N}, \frac{\sum_{j=1}^{N} s_{D2j}}{N},..., \frac{\sum_{j=1}^{N}s_{DCj}}{N}\right] \end{aligned}  $$

In this manner, we can get all the channel importance for the *M* convolutional layers.

Two convolution layers (conv1 and conv2) are selected and the importance of channels in each layer is visualized as Fig. 11(a) and Fig. 11(e). According to the figure, we can see that there are many channels with low importance, which means these channels are redundant and thus can be pruned. In the following, we will detail the channel pruning flow of our scheme.

#### Channel pruning

In work [31], after computing channel weights, the authors then conduct channel pruning by setting a threshold for each layer. More specifically, for a convolutional layer, the following equation is used to determine the pruning threshold,

6 $$ TH = \mu + \sigma + k  $$

where *TH* refers to the pruning threshold, *μ* and *σ* are the mean and the standard deviation of the channel weights in the same layer, respectively. *k* is an adjustable parameter which ranges from 0.1 to 0.5. By setting a lower value to *k*, a higher threshold will be produced and thus more channels will be pruned. We propose another different channel pruning method, which can accurately control how many channels are pruned. Let *O* be the target pruning ratio (say, 50%), and *R* be the number of training loops we want to perform. If equal channel pruning proportion *X* is targeted in each training loop, then we have

7 $$ X + (1-X)X +... (1-X)^{(R-1)}X = O  $$

By solving the above function, we get

8 $$  X = 1 - (1 - O)^{(1/R)}  $$

**Fig. 6:**
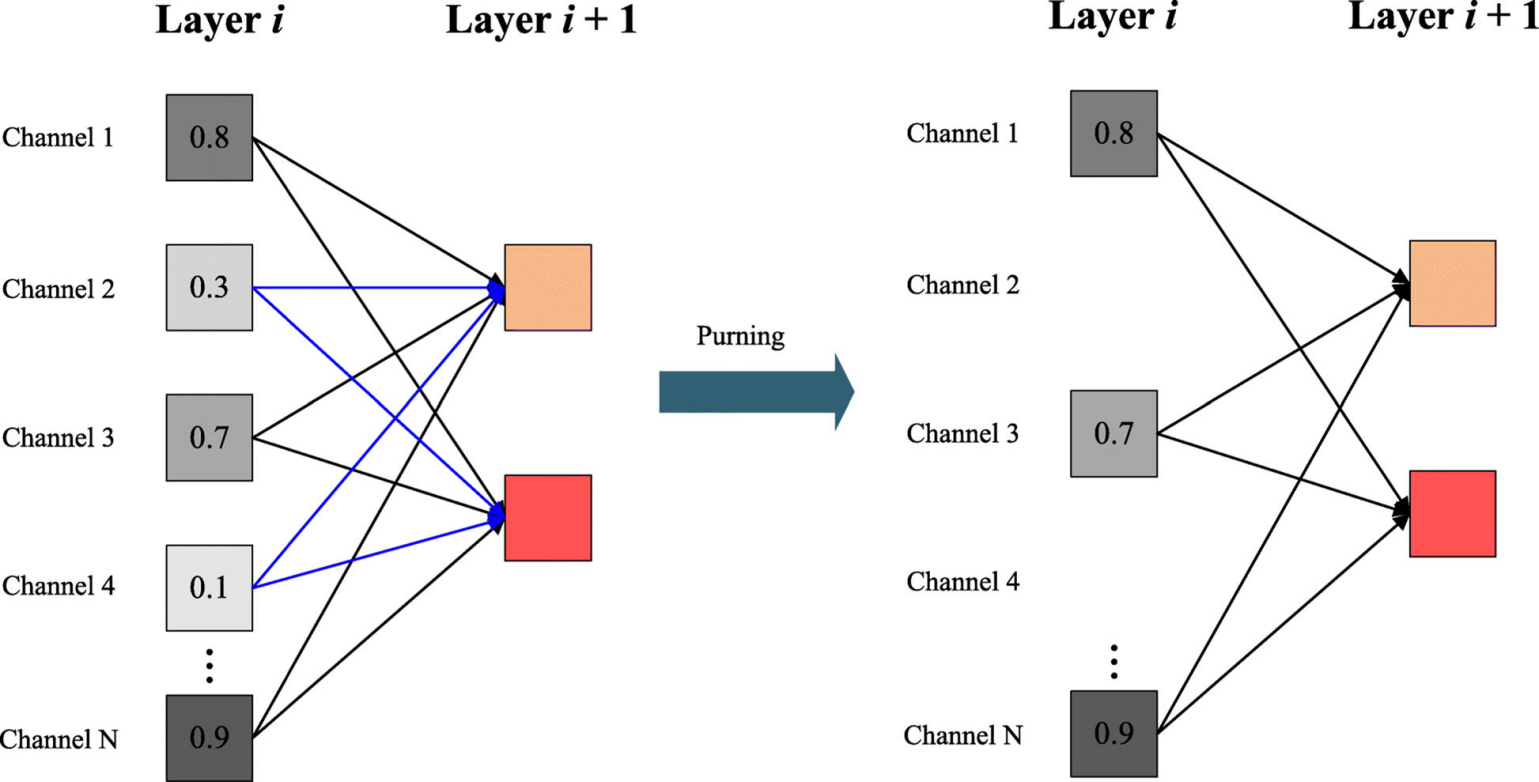
A schematic pruning example. The channels belong to the *X* proportion with low-importance will be pruned

Then in each channel pruning loop, we will discard the unimportant channels which belong to the *X* proportion according to the ranking of weights, as shown in Fig. 6.

### Model assembling scheme

To reduce generalization error and improve performance, multiple hybrid models with the same architecture are assembled together. Each hybrid model is obtained by using a subset of the training data. Our assembling scheme can be treated as a kind of bagging method. Bagging is proposed by Leo Breiman in 1996 [32] to improve classification by combining classifications of randomly generated training sets.

**Fig. 7:**
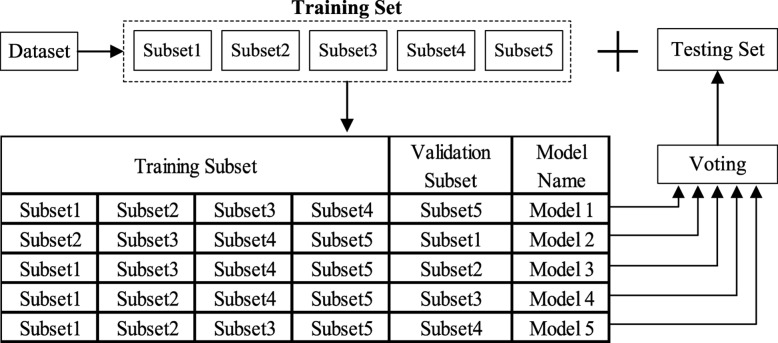
The proposed bagging scheme with five models

As shown in Fig. 7, in this paper we propose a special bagging scheme with 5 models. In detail, the entire dataset is first randomly divided into two parts: a training set and a testing set. The training set is utilized to produce multiple hybrid models, and the testing set is left for evaluating the generation ability of our classification method. The training set is further split into 5 non-overlapping equal subsets with random sampling manner. Four of these subsets are selected as the training samples and the left one subset is chosen as the validation set. Then different classification models can be constructed by using different training and validating set splittings, as shown in Fig. 7. In the inference process, each hybrid model makes a decision and predicts the histology image label. Using a multi-model voting scheme, the final prediction can be produced.

## Results

### Implementation details

The implementation details for our algorithm are presented in this section. Codes and models are available at https://github.com/WendyDong/BreastCancerCNN.

All the experiments are conducted under Centos 7.0 environment. The training process uses 2 NVIDIA GTX 1080Ti 12GB GPUs and adopts the Caffe deep learning framework by the Berkeley Learning and Vision Center (BLVC) [33].

The mini-batch Stochastic Gradient Descent (SGD) method is carried out based on backpropagation and the mini-batch size of 10 is used to update the network parameters, including all the convolution layers and SEP blocks. The initial starting learning rate is 0.0004 and then it decreases exponentially every 10000 iterations. A momentum term of 0.9 and a weight decay of 0.009 are configured in the training process. Our CNN model is trained for 40000 iterations.

### Dataset description

Our method is verified in two breast cancer datasets: BreaKHis and the BreAst Cancer Histology (BACH) [12] dataset.

#### BreaKHis

**Fig. 8:**
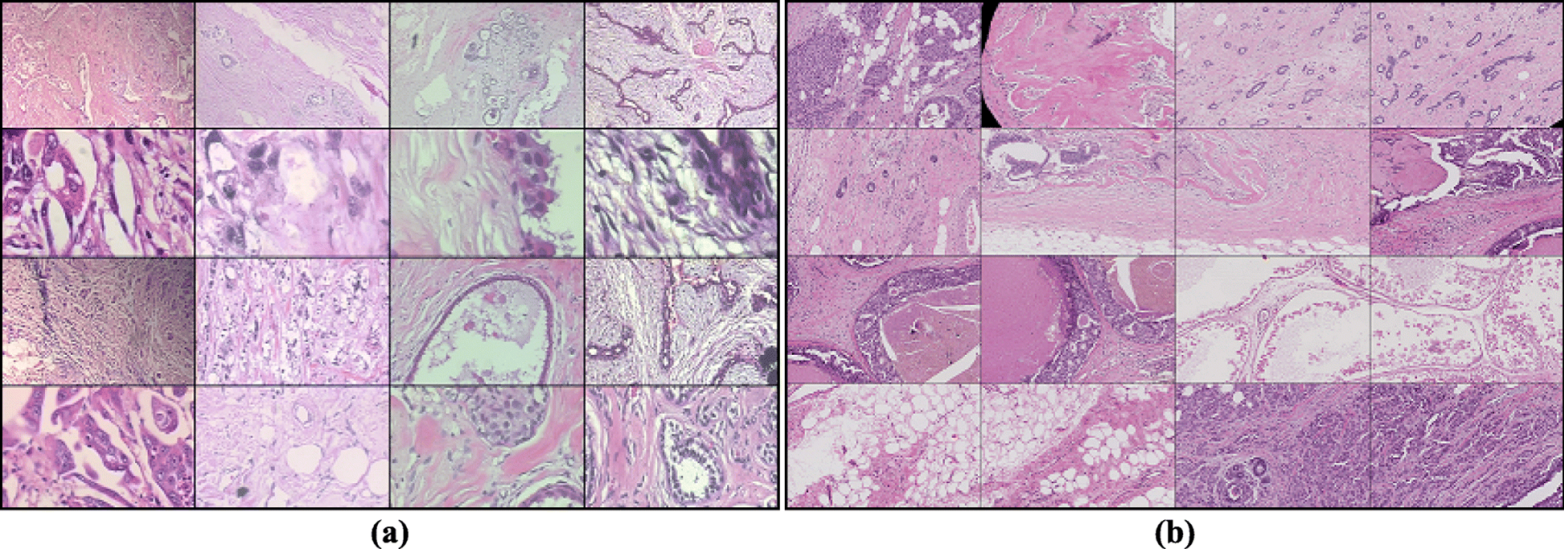
Exemplar images collected from (**a**) BreaKHis dataset and (**b**) BACH dataset

The BreaKHis database is introduced by work [9]. It contains microscopic biopsy images of benign and malignant breast tumors. The database is composed of 7,909 image samples generated from breast tissue biopsy slides, which are stained with HE. The images are divided into benign (adenosis, fibroadenoma, phyllodes tumor, and tubular adenoma) and malignant tumors (ductal carcinoma, lobular carcinoma, mucinous carcinoma, and papillary carcinoma) based on the aspect of the tumoral cells under the microscope. Some exemplar samples are shown in Fig. 8(a).

To ensure a fair comparison, the experimental protocol proposed in [9] is strictly followed. We use the same manner to divide the BreaKHis dataset into training (70%) and testing (30%) set. BreaKHis is mainly used to analyze the classification performance and evaluate the compression strategy of our hybrid model.

#### BACH

The BACH contains 2 types dataset: microscopy dataset and WSI dataset. The BACH microscopy dataset is composed of 400 HE stained breast histology images [34]. All images are of equal dimensions (2048 ×1536), and each image is labeled with one of four classes: (1) normal tissue, (2) benign lesion, (3) in situ carcinoma and (4) invasive carcinoma. The WSI subset consists of 20 whole-slide images of very large size, such as 40000 ×60000. Each WSI can have multiple normal, benign, in situ carcinoma and invasive carcinoma regions. The annotation of the whole-slide images was performed by two medical experts and images where there was disagreement were discarded. Each pixel of these regions (the remaining tissue is considered normal) has a corresponding label indicating benign, in situ carcinoma and invasive carcinoma regions.

In our experiment, BACH WSI dataset is selected to test the algorithm. For each WSI, a series of patches are sampled from multiple key regions, and in Fig. 8(b) some example images are shown. The normal tissue and benign lesion are labeled as the benign class, and in situ carcinoma coupled with invasive carcinoma are treated as cancer lesion. The dataset is divided into a training subset (including validation set) and a testing subset. The training subset is used to train multiple models and the testing subset is adopted to evaluate the performance of our model assembling strategy.

### Evaluation criteria

We report the recognition rate both at the patient level (PL) and the image level (IL) [11]. The patient score (PS) is defined as

9 $$ \rm{PS} = N_{rec}/N_{P}  $$

where *N*_*P*_ is the number of cancer images for patient P and *N*_*rec*_ is the number of images that are correctly classified. Based on PS, the global patient recognition rate is defined as

10 $$ \rm{PL} = \frac{\sum{PS}}{N_{patient}}  $$

where *N*_*patient*_ is the number of the patient.

The image level recognition rate is calculated by the following function,

11 $$ \rm{IL} = \frac{N_{rec}}{N_{all}}  $$

where *N*_*all*_ is the number of cancer images of the test set and *N*_*rec*_ is the correctly classified cancer images.

Besides, we also include positive predictive value (PPV) and Cohen’s Kappa for further evaluation:

12 $$ \rm{PPV} = \frac{TP}{TP+FP}  $$

where TP, TN, FP, and FN represent true positives, true negatives, false positives, and false negatives, respectively.

13 $$ \rm{Kappa} = \frac{Acc-Acc_{r}}{1-Acc_{r}}   $$

where *Acc*= (*TP*+*TN*)/(*TP*+*TN*+*FP*+*FN*). In this work, Kappa measures the agreement between the machine learning scheme and the human ground truth labeled by pathologists. In (13), *Acc* is the relative observed agreement, and *Acc*_*r*_ is is the hypothetical probability of chance agreement, which can be computed as the probability of each classifier randomly selecting each category by using the observed data [35].

### Classification results

Classification results of three methods are listed to fully evaluate the contributions of each part in our model: 1. results based on only the global model branch; 2. results based on only the local model branch; 3. results based on the proposed hybrid CNN model. For method 1, each input image is directly processed by the global model. For method 2, 15 non-overlapping patches are extracted from each input image and then they are put into the local model generating 15 prediction results. Then voting is performed to classify the input image based on the average of 15 predictions. For method 3, both local branch and global branch predictions are merged together by (1) to generate the final results (0.6 is selected for *λ* in our experiment). Besides, we also show the results of using majority voting (*Max*) scheme when merging patch predictions, denoted as “2(*Max*)" and “3(*Max*)" in the table.

The results of the above methods are shown in Table 2 and Table 3 in terms of accuracy, Kappa and PPV on both BACH and BreaKHis. Similar to work [11], both patient and image level results are calculated for accuracy. Besides, F1 score, sensitivity, and precision for image level performance is further discussed on BreaKHis, as shown in Table 4.

**Table 2:** Classification Results on BACH

Str.	IL(Acc.)	PL(Acc.)	Kappa	PPV
1	86.2 ±1.9	82.3 ±3.4	0.724 ±0.037	84.2 ±2.8
2(Max)	84.8 ±2.4	82.3 ±2.6	0.697 ±0.048	87.2 ±3.2
2	84.8 ±2.3	82.6 ±2.3	0.695 ±0.046	83.5 ±3.2
3(Max)	86.4 ±1.5	84.1 ±1.3	0.727 ±0.030	88.5 ±1.67
3	86.6 ±1.7	83.1 ±1.7	0.732 ±0.033	84.7 ±2.48

**Table 3:** Classification Results on BreaKHis

Cri.	Str.	Magnification Factors			
		40 ×	100 ×	200 ×	400 ×
PL(Acc.)	1	82.4 ±3.4	80.8 ±1.1	81.3 ±1.5	77.3 ±2.9
	2(Max)	83.7 ±2.3	81.4 ±2.9	82.8 ±3.7	79.0 ±4.6
	2	83.9 ±2.3	82.2 ±3.7	83.4 ±1.8	79.6 ±5.0
	3(Max)	83.8 ±2.3	82.3 ±1.6	83.5 ±2.5	79.2 ±4.8
	3	84.5 ±2.5	83.4 ±2.5	83.9 ±1.7	80.0 ±4.3
IL(Acc.)	1	82.0 ±2.5	81.1 ±0.9	81.4 ±1.8	76.8 ±3.9
	2(Max)	84.3 ±0.9	81.5 ±3.1	84.0 ±4.6	79.7 ±4.4
	2	85.0 ±1.3	83.6 ±3.1	84.6 ±1.8	80.4 ±5.1
	3(Max)	84.8 ±0.9	82.7 ±1.7	84.7 ±3.5	79.8 ±4.6
	3	85.6 ±1.4	83.9 ±2.8	85.4 ±1.4	81.2 ±4.5
Kappa	1	0.585 ±0.050	0.547 ±0.031	0.563 ±0.020	0.449 ±0.090
	2(Max)	0.635 ±0.030	0.536 ±0.123	0.619 ±0.119	0.500 ±0.132
	2	0.637 ±0.036	0.525 ±0.131	0.607 ±0.102	0.514 ±0.149
	3(Max)	0.635 ±0.029	0.579 ±0.061	0.637 ±0.085	0.504 ±0.135
	3	0.651 ±0.039	0.551 ±0.106	0.625 ±0.087	0.535 ±0.128
PPV	1	75.1 ±6.2	77.4 ±4.8	73.4 ±5.7	70.4 ±7.4
	2(Max)	84.7 ±3.8	81.4 ±2.7	78.5 ±0.7	77.3 ±5.3
	2	85.9 ±3.5	81.6 ±1.9	79.4 ±1.7	79.5 ±6.6
	3(Max)	84.7 ±3.9	82.1 ±1.5	79.2 ±1.2	77.8 ±5.5
	3	86.4 ±2.4	83.3 ±2.1	80.1 ±2.1	81.3 ±5.1

**Table 4:** F1, precision, and recall on BreaKHis

Cri.	Str.	Magnification Factors			
		40 ×	100 ×	200 ×	400 ×
F1	1	86.7 ±2.2	86.6 ±0.6	86.4 ±2.1	83.5 ±2.9
	2	89.4 ±0.8	88.6 ±1.7	89.0 ±1.7	86.3 ±3.2
	3	89.8 ±1.0	88.8 ±1.5	89.4 ±1.3	86.8 ±2.8
Pr.	1	85.4 ±1.7	82.5 ±0.9	85.2 ±2.1	79.2 ±3.7
	2	84.7 ±1.7	83.7 ±5.1	87.5 ±2.3	81.2 ±5.7
	3	85.3 ±1.8	83.9 ±4.7	88.0 ±1.5	81.6 ±5.1
Rec.	1	88.3 ±4.0	91.3 ±2.1	87.7 ±4.8	88.5 ±3.3
	2	94.8 ±1.2	94.5 ±3.0	90.6 ±2.0	92.5 ±3.2
	3	94.9 ±0.8	94.6 ±2.9	90.8 ±1.9	93.1 ±3.0

As can be seen from Table 2 and Table 3, method 1 has already produced a decent accuracy by using the global branch model. In most cases of Table 2 and Table 3, some improvements can be observed for the local branch model voting strategy (method 2) when compared to the global branch model. Although method 2 can achieve comparable performances with method 3 for some cases, such as the IL results of BreakHis 40×, as shown in Table 3. However, there are still many cases that the hybrid model achieves obviously better results than the local voting scheme. On the whole, the hybrid model (method 3) achieves the best result among all the three methods. This means that the local information and global information can effectively work together to make the decision. In fact, although the patch-level voting scheme in method 2 gives some cue for the global-level information, the global branch model of method 1 can extract stronger effective global representation when processing the input image as a whole. Besides, for different magnification factors, the recognition algorithm (such as method 3) produces different performances. On 40 × and 200 × datasets, higher accuracy is prone to happen when compared to 100 × and 400 × datasets.

**Fig. 9:**
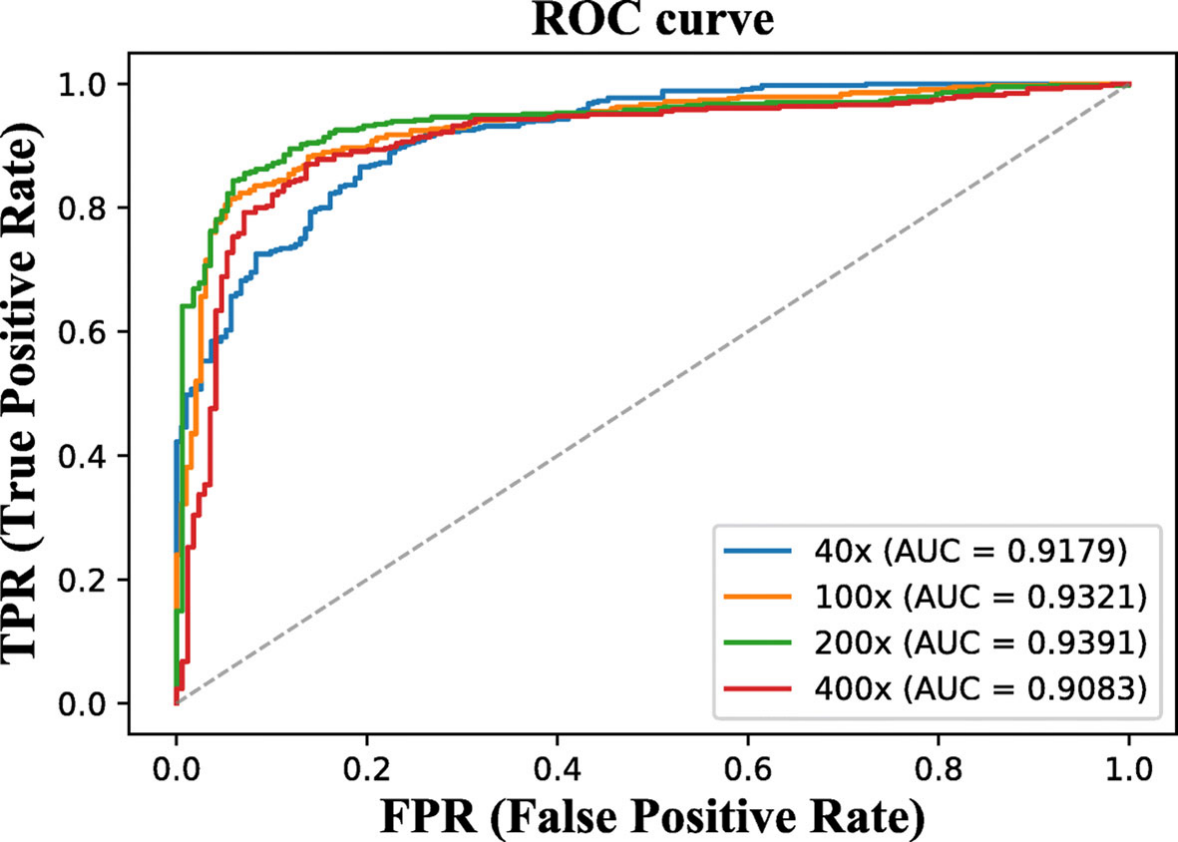
RoC curves of our hybrid model in different magnification factors

From Table 4, one can notice that the similar phenomenon happens to F1 score, sensitivity and precision for our methods: local branch voting strategy achieves higher performance than global branch; hybrid model produces the optimal results. The performance of our hybrid model is further analyzed by drawing the associated ROC curve, as shown in Fig. 9. The 200 × magnification factor shows the best results among performances obtained with different magnification levels under 0.4 False Positive Rate (FPR). However, when FPR is higher than 0.4, the 40 × magnification factor produces a superior performance to 200 ×. Overall, 200 × magnification factor shows a higher potential than the other magnification factors.

### Compact model performance

In this paper, we set the specific target pruning ratio *O*=50*%*, and let the training loops *R*=1. According to (8), 50% channels should be removed in one pruning process. With 50% channel pruning, accuracy, F1 score, sensitivity and precision are listed in Table 5 and Table 6. The optimized compact hybrid model achieves comparable results when compared with Table 3 and Table 4. Some results in Table 5 and Table 6 even slightly outperforms the original model, such as 40 × and 100 ×. The possible reason is that the compact model has a lower risk of overfitting by removing some redundancy.

**Table 5:** Classification results after pruning 50% channels

Acc.	Str.	Magnification Factors			
		40 ×	100 ×	200 ×	400 ×
PL	1	82.4 ±3.5	80.2 ±9.5	81.9 ±5.4	75.7 ±3.3
	2	84.9 ±2.5	83.1 ±3.9	84.0 ±1.3	79.3 ±5.1
	3	85.2 ±2.6	83.5 ±3.8	84.1 ±1.4	79.3 ±2.7
IL	1	81.3 ±2.9	79.9 ±0.8	81.7 ±1.3	75.3 ±3.5
	2	85.2 ±1.7	83.8 ±2.9	84.8 ±1.8	80.2 ±5.0
	3	85.7 ±1.9	84.2 ±3.2	84.9 ±2.2	80.1 ±4.4

**Table 6:** F1, precision, and recall after pruning 50% channels

Cri.	Str.	Magnification Factors			
		40 ×	100 ×	200 ×	400 ×
F1	1	86.9 ±1.7	86.0 ±0.7	87.1 ±1.2	82.5 ±2.3
	2	89.9 ±1.1	88.5 ±1.4	89.1 ±1.7	86.1 ±3.1
	3	90.0 ±1.2	88.8 ±1.7	89.2 ±1.9	86.1 ±2.7
Pr.	1	82.8 ±4.2	80.9 ±2.2	83.9 ±3.4	78.2 ±4.1
	2	84.6 ±2.2	85.4 ±5.1	86.4 ±2.2	81.5 ±5.6
	3	84.6 ±2.5	85.2 ±5.1	86.1 ±2.8	81.0 ±5.0
Rec.	1	91.7 ±2.5	91.8 ±2.5	90.8 ±3.3	87.4 ±1.2
	2	95.9 ±0.7	92.2 ±3.4	92.0 ±2.2	91.6 ±3.9
	3	96.3 ±0.9	93.2 ±2.9	92.7 ±2.3	92.2 ±3.2

**Fig. 10:**
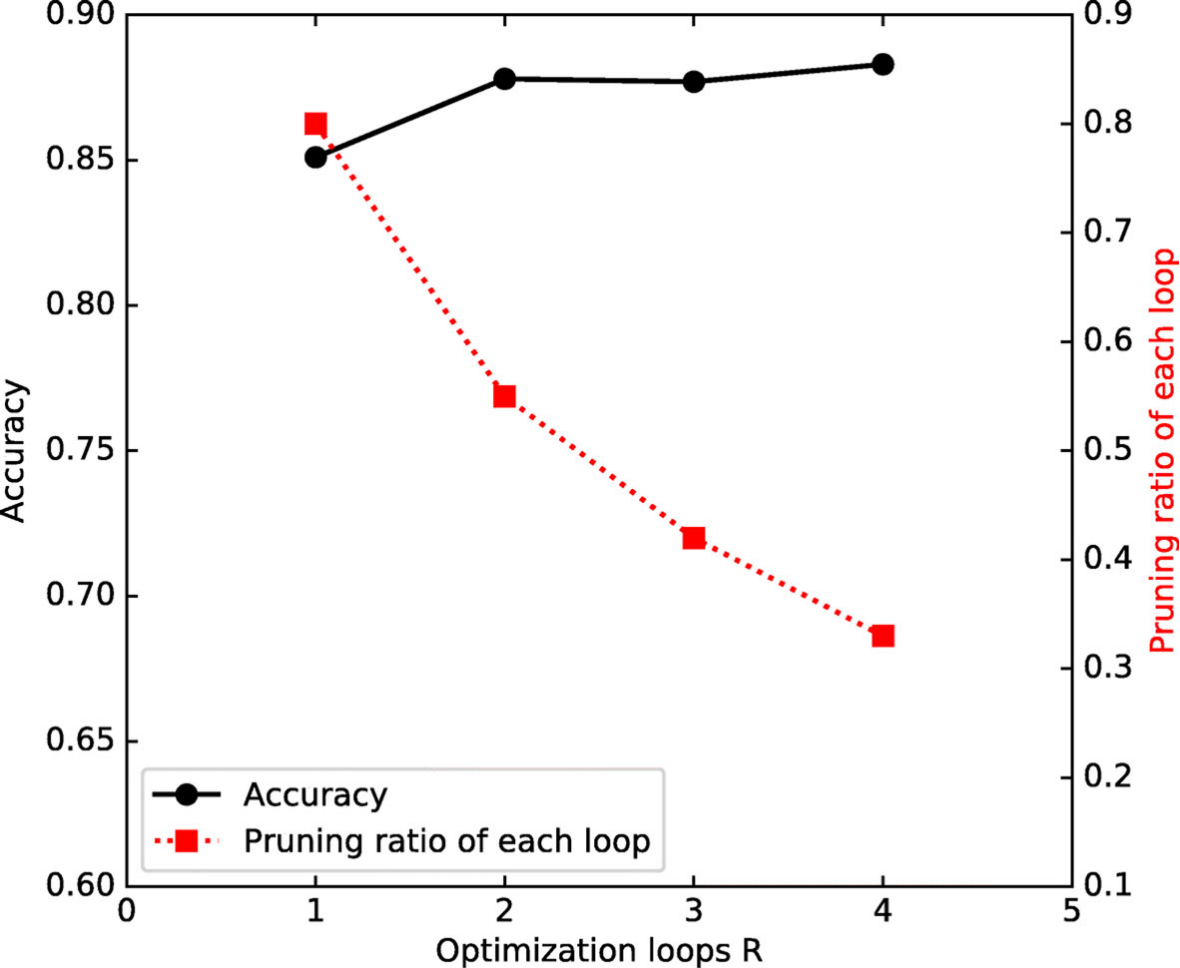
A channel-pruning example with target pruning ratio 80%. The black line represents the compressed model accuracy [0.851,0.878,0.877,0.883] with *R* from 1 to 4; the red dotted line denotes the corresponding pruning proportion *X* [0.8,0.55,0.42,0.33] for each loop under 4 different situations

In Fig. 10, a channel pruning example with different *R* (1 to 4) under the same target pruning ratio *O*=80*%* is shown to further analyze the relationship between accuracy and *R*. With the increasing of *R*, the model accuracy is improved accordingly and the pruning proportion *X* for each loop drops. This tells that by increasing training loops *R* our model performance will be further improved slightly, but more training loops (computing resources) will be needed. In our experiment, we already can achieve decent results by setting training loops *R*=1.

**Fig. 11:**
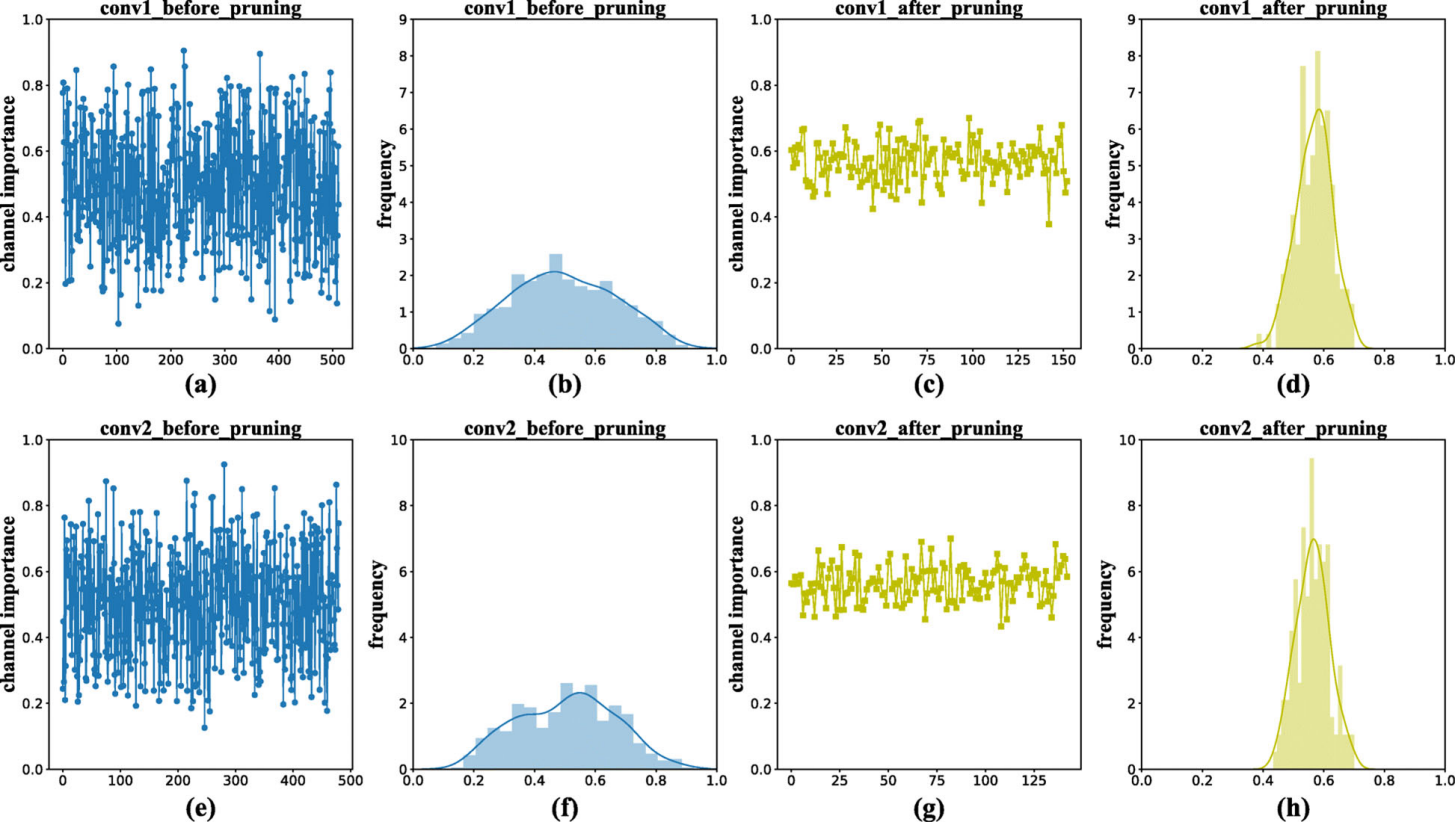
Channel pruning visualization of two convolution layers. (**a**) (**e**): The original importance distributions before channel pruning. (**b**) (**f**): Histograms of original importance distributions. (**c**) (**d**): The importance distributions after channel pruning. (**g**) (**h**): Histograms of importance distributions for the pruned network

In Fig. 11, the distributions of channel importance of the two selected channels are also visualized after pruning. We can see that the channel importances have more compact distribution (with lower variance) and almost all remaining channels have equal importance value (around 0.5). This means that all the selected channels have sufficient information and no channel is obviously superior to the others.

**Fig. 12:**
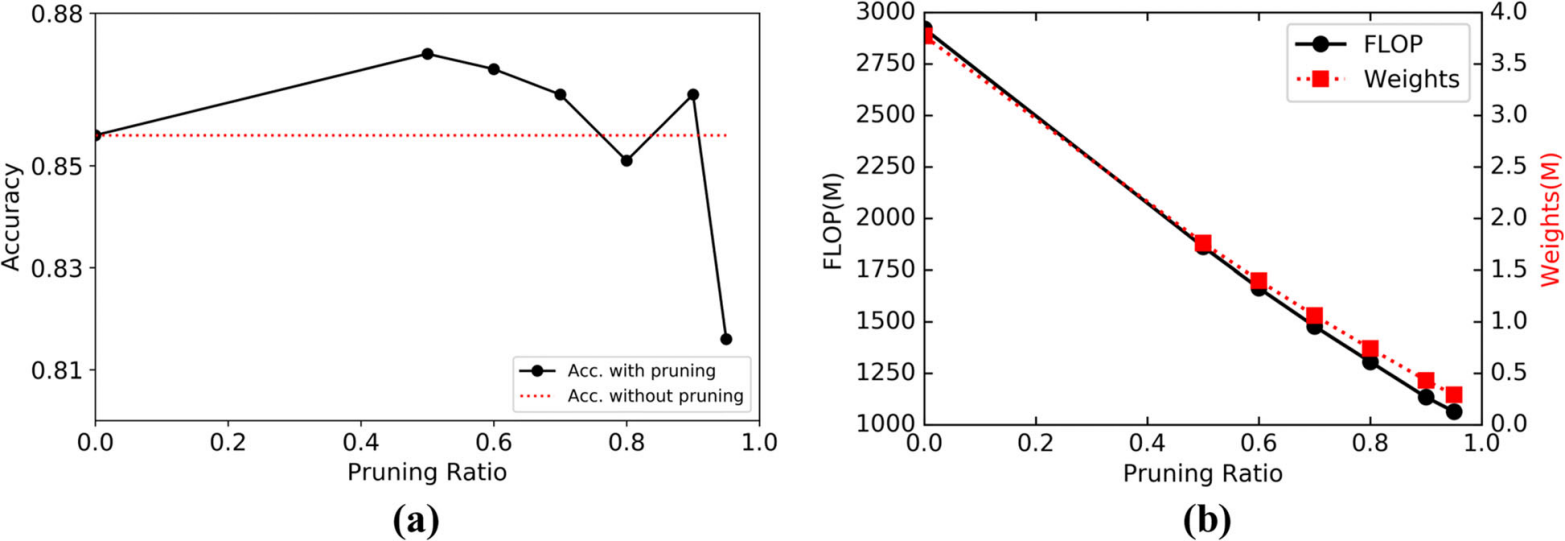
Classification accuracy, FLOPs and weights under different pruning ratios

We also analyze the relationship between accuracy and different pruning ratios of our compact model. By choosing a model trained by 40 × dataset, the performance with different pruning ratios is depicted in Fig. 12(a). From the figure, one can see that under a certain pruning ratio threshold (say, 90%), the pruned network produces comparable accuracy (actually most points perform better) with the original model. However, it will ruin the accuracy when the pruning ratio increases further. For example, the accuracy will drop sharply to 0.816 with 95% pruning ratio. Under different pruning ratios, the float-point-operations (FLOPs) and weights are also depicted in Fig. 12(b). The number of FLOPs and weights almost decreases linearly. It is worth noting that the declining speed of FLOPs and weights will slow down when the pruning ratio is close to 1. The reason is that the first three convolution layers are not pruned (without flowed SEP blocks) in our hybrid model as denoted in Fig. 2. For clarity, the results in Fig. 12(b) are also tabulated as Table 7 to show the model size and FLOPs improvement by using our method. The weights and FLOPs of work [11] and [17] are also included in Table 7. With the increase of pruning ratio, our model will have the smallest amount of weights.

**Table 7:** Weights size and FLOPs improvement by using our channel pruning scheme under different pruning ratios

Method	Pruning Degree	Weights (M)	FLOP (M)
Our	Before Pruning	3.77	2920.3
	Pruning Ratio 0.5	1.76	1861.8
	Pruning Ratio 0.6	1.4	1663.1
	Pruning Ratio 0.7	1.06	1477.8
	Pruning Ratio 0.8	0.74	1305
	Pruning Ratio 0.9	0.43	1133.6
	Pruning Ratio 0.95	0.29	1063.8
Work[11]	N/A	0.55	47.4/188.5
Work[17]	N/A	13.5	8521

The weights and FLOPs of work [11] and [17] are also included in the table. The work [11] has two types of networks with different input sizes: 32 ×32 and 64 ×64, and the corresponding FLOPs are 47.4 (M) and 188.5 (M), respectively

**Fig. 13:**
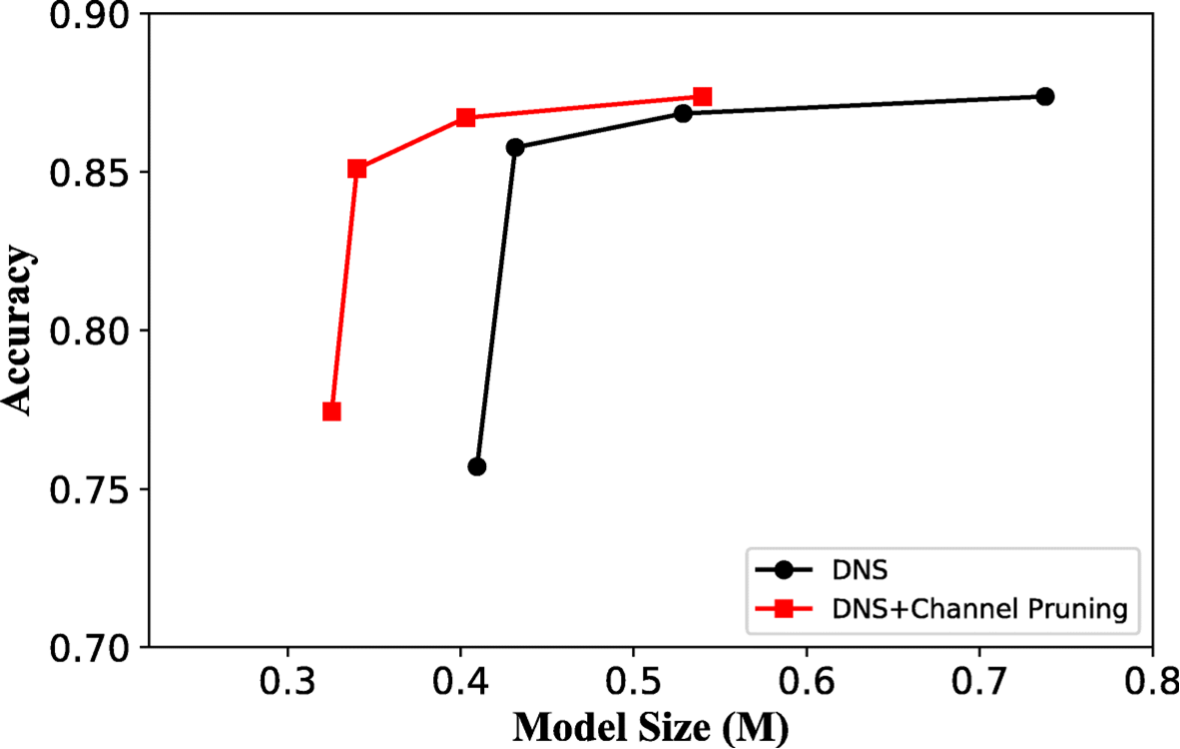
Classification accuracy by combining different model compression schemes

To make the model more compact, the other traditional compression scheme Dynamic Network Surgery (DNS) [25] method, which can properly incorporate connection splicing into the training process to avoid incorrect pruning, is merged with our method. The result in Fig. 13 shows the recognition accuracies by using our channel pruning and DNS together. From the figure we can see that the joint approach far outperforms the results only using DNS, especially in the small model size range.

### Performance comparisons

For BreaKHis dataset, the results reported in related works are the average of five trials, and the folds are provided along with the dataset to allow for a full comparison of classification results [9]. For the fair comparison, the same dataset partition and fold segmentation are used in our test. However, it should be noted that the multi-model assembling scheme requires dividing the dataset into training subsets, validation subsets and testing dataset, which needs different data partition manner with the BreaKHis dataset. Thus, we just compare our method without the multi-model assembling technique to the other works for BreakHis dataset. To show the performance comparisons of our complete scheme with the other works, the testing is performed on the samples from BACH WSI dataset. In detail, 10270 images of size 512×768 are sampled, 2645 of which are used as the testing dataset and the left 7625 samples are adopted to train multiple (5 models are generated in our experiment) models. For each specific model (each fold), 6100 samples are as training pictures and 1525 samples are utilized for validation, according to our bagging scheme. For each samples of the 6100 training data, 8 pictures are generated according to our data augmentation method. After data augmentation, each image is resized to 1120×672. Then 15 non-overlapping patches with size 224×224 are extracted from each image. Therefore, totally 6100×8 images and 6100×8×15 patches are generated for each fold. The 6100×8 images are used to train the global branch and the 6100×8×15 patches are used to train the local branch of the model.

In Table 8, we list the result of our hybrid model without multi-model assembling together with the experimental results presented in [9], [17] and [11]. All the reported results in work [17] are patient level and the results of image level are not available. All works listed for comparison are strictly following the data partition manner in work [9]. As presented in Table 8, work [11] achieves the best patient accuracy among all the magnification factors. Our hybrid model achieves the second place for 40× and 100× magnification factors. For image level testing, our hybrid model gets slightly better results for 40×, 100× and 200× factors when compared to work [11]. In work [11], the reported results are obtained by combining four patch-level models trained with different patch generation strategies, which produces the state-of-the-art for patient level result. In the following, we will compare the proposed hybrid model coupling with our model assembling technique to work [11].

**Table 8:** Performance comparisons between our hybrid model and the other schemes on BreaKHis

Acc.	Str.	Magnification Factors			
		40 ×	100 ×	200 ×	400 ×
PL	[9]	83.8 ±4.1	82.1 ±4.9	85.1 ±3.1	82.3 ±3.8
	[17]	83.0 ±3.0	83.2 ±3.5	84.6 ±2.7	82.1 ±4.4
	[11]	90.0 ±6.7	88.4 ±4.8	84.6 ±4.2	86.1 ±6.2
	Our	85.2 ±2.6	83.5 ±3.8	84.1 ±1.4	79.3 ±2.7
IL	[9]	82.8 ±3.6	80.7 ±4.9	84.2 ±1.6	81.2 ±3.6
	[11]	85.6 ±4.8	83.5 ±3.9	83.1 ±1.9	80.8 ±3.0
	Our	85.7 ±1.9	84.2 ±3.2	84.9 ±2.2	80.1 ±4.4

In work [11], the authors provide two strategies to generate the training samples: sliding window allowing 50% of overlap between patches; random extraction strategy with a fixed arbitrary number of patches (such as 1000) from each input image. Besides, the authors use 2 patch sizes for each strategy (32×32 and 64×64), and thus totally 4 different models are generated based on different training set. We reproduce the 4 models and use *Max* rule (which shows higher accuracy than *Sum* and *Product* rules in [11]) to merge them. For our work, 5 models are trained and assembled together using *Sum* rule to vote for the final image label. Table 9 summarizes the comparisons between our work and different schemes in work [11]. Sliding window scheme of 64×64 achieves the best performance among all the 4 patch models of work [11], which produces 82.1*%* PL and 77.1*%* IL, respectively. By using the *Max* merging scheme, the recognition accuracy can be improved to 85.1*%* and 79.3*%*, respectively. By adopting the multi-model assembling strategy, our method can achieve 87.5*%* patient level and 84.4*%* image level accuracy, which outperforms the best results of work [11].

**Table 9:** Performance comparison between our scheme (with assembling) and the state-of-the-art work [11] on BACH

Methods	Strategy	PL	IL	Kappa	PPV
Work [11]	32 ×32 (random sampling)	80.5	76.8	0.608	78.7
	64 ×64 (random sampling)	79.9	74.8	0.595	76.5
	32 ×32 (sliding window)	80.4	75.5	0.607	76.7
	64 ×64 (sliding window)	82.1	77.1	0.641	76.2
	Max Fusion	85.1	79.3	0.700	78.4
This work	Hybrid model with assembling (Sum)	87.5	84.4	0.749	85.7
	Hybrid model with assembling (Max)	87.4	84.2	0.748	84.6

## Discussion

In this study, a breast cancer histopathology image classification by assembling multiple compact CNNs is proposed. Compared to reported breast cancer recognition algorithms that are evaluated on the publicly available BreaKHis dataset, our proposed hybrid model achieves comparable or better performance (see Table 8), indicating the potential of combing both local model and global model branches. By embedding the SEP block into our hybrid model, the channel importance can be learned and the redundant channels are then removed. Under a certain amount of channel pruning, the optimized compact network even produces better performance than the original model, which confirms that the model compression technique can lower the risk of overfitting (see Table 5). However, over pruning channels (say pruning 95%) may harm the model performance largely (see Fig. 12(a)). We also show that our channel pruning scheme can be used in conjunction with the other traditional compression methods, such as DNS in work [25], and this will generate higher accuracy with the same model size (see Fig. 13). The evaluation on the BACH dataset shows that the proposed hybrid model with multi-model assembling scheme outperforms the state-of-the-art work [11] in both patient level and image level accuracy. Actually, we have verified the effectiveness of our model assembling strategy in BACH challenge [34, 36], which is held as part of the ICIAR 2018. It suggests that model assembling is crucial to the task of breast cancer image (which has large variability in morphology) classification and can enhance the model generalization ability, especially in small dataset situation.

The application of machine learning technology, especially deep learning, to medical area research has become more and more popular recently. The significance of the machine learning algorithms is that it can reduce the workload of pathologists, improve the quality of diagnosis, and reduce the risk of misdiagnosis. Our proposed scheme in this work can be used in breast cancer auxiliary diagnostic scenario, and realize workload reducing and diagnosis quality promoting talked above. The first objective of this paper is still to ensure accuracy like the other works, and we propose hybrid architecture and model assembling to achieve this goal. Under the premise of guaranteeing this, we have introduced a channel pruning scheme to make our model more compact, which reduces the computing burden. It should be noted that this study has only proposed and analyzed a channel-level pruning scheme for our hybrid model, and we do not target maximizing the model compression. If targeting higher model compression, the other model compression algorithms should be used together.

In the future, we will involve the experience of the pathologists to guide our model design. Through visualizing deep neural network decision [37], we will try to highlight areas in a given input breast cancer image that provide evidence for or against a certain tumor type. Then, we could find out the differences of supporting areas when making decision between pathologists and algorithms. In addition, by applying the diagnostic experience as a priori, we target constructing an attention-based model and thus improve the accuracy of our model in future work.

## Conclusion

We have proposed breast cancer histopathology image classification based on assembling multiple compact CNNs. The proposed scheme achieves promising results for the breast cancer image classification task. Our method can be used in breast cancer auxiliary diagnostic scenario, and it can reduce the workload of pathologists as well as improve the quality of diagnosis.

## Abbreviations

BACH: Breast cancer histology
BN: Batch normalization
CNNs: Convolutional neural networks
DeCAF: Deep convolutional activation feature
DNS: Dynamic network surgery
FC: Fully connected
FLOPs: Float point operations
FN: False negatives
FP: False positives
GPU: Graphics processing unit
HE: Hematoxylin and eosin
IL: Image level
LBP: Local binary patterns
LPQ: Local phase quantization
PL: Patient level
PPV: Positive predictive value
SEP: Squeeze excitation pruning
SVM: Support vector machines
TN: True negatives
TP: True positives
WSI: Whole slide images
